# A maladaptive ER stress response triggers dysfunction in highly active muscles of mice with SELENON loss

**DOI:** 10.1016/j.redox.2018.10.017

**Published:** 2018-10-26

**Authors:** Diego Pozzer, Ersilia Varone, Alexander Chernorudskiy, Silvia Schiarea, Sonia Missiroli, Carlotta Giorgi, Paolo Pinton, Marta Canato, Elena Germinario, Leonardo Nogara, Bert Blaauw, Ester Zito

**Affiliations:** a Dulbecco Telethon Institute at Istituto di Ricerche Farmacologiche Mario Negri IRCCS, Milan, Italy; b Section of Pathology, Oncology and Experimental Biology, Laboratory for Technologies of Advanced Therapies (LTTA), Department of Morphology, Surgery and Experimental Medicine, University of Ferrara, Ferrara, Italy; c Maria Cecilia Hospital, GVM Care & Research, 48033 Cotignola, Ravenna, Italy; d Department of Biomedical Sciences, University of Padua, Padua, Italy; e Venetian Institute of Molecular Medicine, Padua, Italy

**Keywords:** Diaphragm dysfunction, ER stress response, SELENON

## Abstract

Selenoprotein N (SELENON) is an endoplasmic reticulum (ER) protein whose loss of function leads to human SELENON-related myopathies. *SelenoN* knockout (KO) mouse limb muscles, however, are protected from the disease, and display no major alterations in muscle histology or contractile properties. Interestingly, we find that the highly active diaphragm muscle shows impaired force production, in line with the human phenotype. In addition, after repeated stimulation with a protocol which induces muscle fatigue, also hind limb muscles show altered relaxation times. Mechanistically, muscle SELENON loss alters activity-dependent calcium handling selectively impinging on the Ca^2+^ uptake of the sarcoplasmic reticulum and elicits an ER stress response, including the expression of the maladaptive CHOP-induced ERO1. In SELENON-devoid models, ERO1 shifts ER redox to a more oxidised poise, and further affects Ca^2+^ uptake. Importantly, CHOP ablation in *SelenoN* KO mice completely prevents diaphragm dysfunction, the prolonged limb muscle relaxation after fatigue, and restores Ca^2+^ uptake by attenuating the induction of ERO1. These findings suggest that SELENON is part of an ER stress-dependent antioxidant response and that the CHOP/ERO1 branch of the ER stress response is a novel pathogenic mechanism underlying SELENON-related myopathies.

## 1 Introduction

SELENON-related myopathies (SELENON-RM) are a group of muscle diseases due to homozygous loss of function mutations in *SELENON* gene (previously called *SEPN1*) and characterised by a heterogenous spectrum of clinical features. Spinal rigidity and respiratory impairment due to weakness in diaphragm muscle are features of the most commonly observed SELENON-RM phenotypes [11], [28], [6].

A major obstacle when treating SELENON-RM patients is that little is known about SELENON function, partly because of the difficulty in obtaining the pure protein necessary to test its activity. However, despite this, it has been established that SELENON has an endoplasmic/sarcoplasmic reticulum (ER/SR) localisation and a thioredoxin reductase-like domain on its ER side [32].

Some overlapping phenotypic signs of the pathogenic mutations in *SELENON* and the calcium release channel, *RyR1* have prompted studies aimed at characterising the functional interaction of SELENON and RYR1 [18].

Using an unbiased proteomic approach, we have recently shown that redox-active *SELENON* interacts with the SR/ER Ca^2+^ pump, SERCA2, and measurements of Ca^2+^ transients in the ER of *SELENON-*devoid eukaryotic cells indicate that SERCA activity is impaired [27].

Skeletal muscle is sensitive to the physiological triggers of the ER stress response/unfolded protein response (UPR), including unbalanced Ca^2+^ homeostasis and hypoxia [1] and the UPR is possibly involved in skeletal muscle function during physiologically challenging conditions [44]. However, in some circumstances its persistence can be maladaptive, and in fact deletion of the gene encoding CHOP (CAATT enhancer-binding protein homologous protein, a transcription factor active in the UPR) protects cells against apoptosis during ER stress [26].

SELENON levels are responsive to ER stress and are co-regulated with those of the CHOP-induced ER stress mediator, disulphide oxidase endoplasmic oxidoreductin 1 (ERO1), high levels of which have also been associated with negative outcomes of the ER stress response [15], [26], [27]. Functional interactions between SELENON and ERO1 have been suggested by inducing a myopathic phenotype through the delivery of an ERO1-containing adeno-associated virus to otherwise normal SELENON KO limb muscles. Furthermore, the increased cell survival observed after treating SELENON KO cells with an ERO1 inhibitor is consistent with a role of SELENON in counteracting the effects of ERO1 [27], [35].

In this study, we show that SELENON loss impinges on muscle performance in an activity-dependent manner. Limb muscles from SELENON KO mice are spared from myopathy, however they showed impaired relaxation after an exercise mimetic of tetanic stimulation. Importantly, the more active diaphragm muscle is affected at an early age, as documented by impaired force and an increased maladaptive ER stress response. The ablation of the maladaptive ER stress response mediator CHOP, upstream to ERO1, completely prevents diaphragm weakness, the impaired force relaxation in the limb muscles and restores Ca^2+^ transients by reducing ERO1 levels in SELENON KO mice. These results indicate that SELENON may have evolved as part of an ER stress-dependent anti-oxidant response in active muscles, and that the CHOP-ERO1 branch of the ER stress response provides a novel pathogenic mechanism underlying SELENON-related myopathies.

## 2 Results

### 2.1 Hypoxic conditions elicit an exacerbated response of the ER stress mediators ERO1 and CHOP in SELENON-devoid cells

ER stress response is a conserved ancient pathway initiated by ER stress conditions that impede protein folding. Usually, ER stress response promotes proteostasis through induction of chaperones and the attenuation of protein translation by three different arms: IRE-1, PERK and ATF6. IRE-1 splices the mRNA of the transcription factor X-box-binding protein 1 (XBP-1 s), which activates transcription of ER stress response target genes. PERK attenuates protein translation and upregulates the transcription factors ATF4 and CHOP with the downstream protein disulphide oxidase ERO1 alpha (henceforth ERO1) and the phosphatase GADD34. ATF6 promotes the induction of BIP and other chaperones [37].

In order to test ER stress responses in SELENON-deficient cells, we used SELENON KD C2C12 cells [27], [35].

**Fig. 1:**
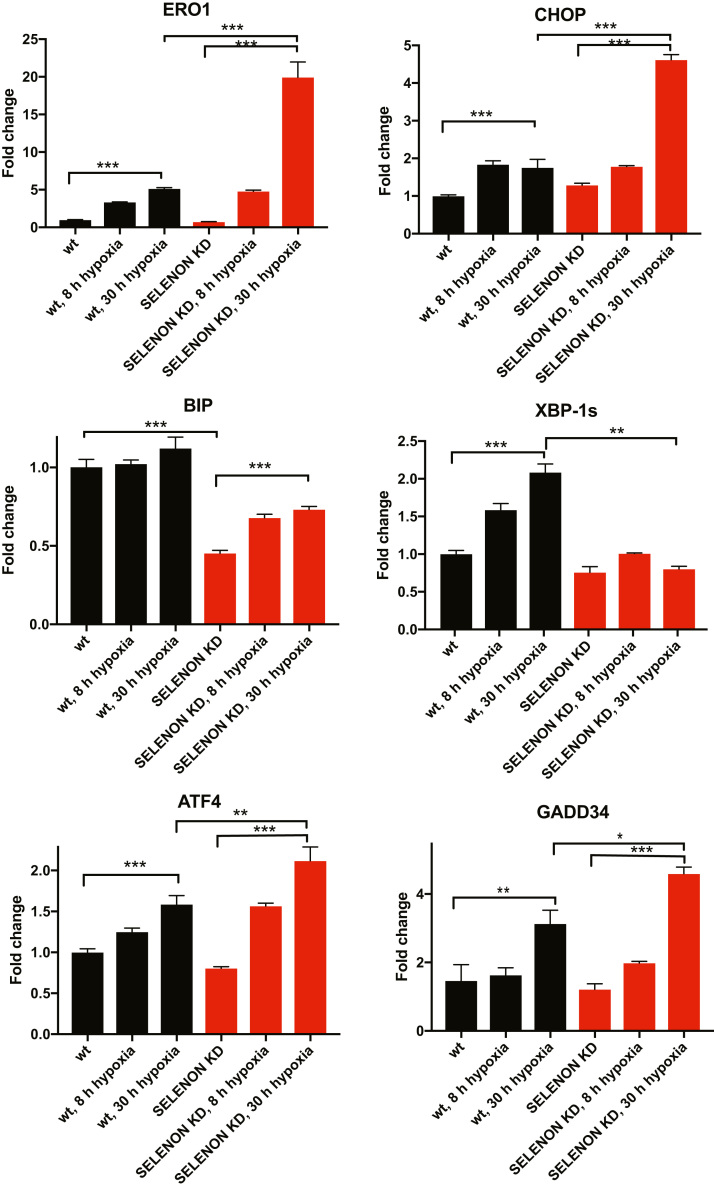
Hypoxia elicits an exacerbated ER stress response with a prominent increase in CHOP and its target ERO1 in SELENON depleted myoblasts. A) Semi-quantitative, real-time RT-PCR analysis of ER stress response markers in mRNA prepared from wild-type (WT) and SELENON KD C2C12 cells exposed to hypoxia for the indicated periods of time (hours, h) (n = 4).

Surprisingly, mRNA levels of molecular components of the anti-adaptation branch of the ER stress response (as CHOP, ERO1, ATF4 and GADD34) were induced in SELENON KD C2C12 cells. However, ERO1 and its upstream regulator CHOP were more upregulated, whereas the mRNA levels of the pro-adaptive marker BIP was reduced under hypoxic conditions in KD cells (Fig. 1). Of note, we did not detect higher levels of ERO1 and CHOP in SELENON KD cells after treatment with the common ER stressors, tunicamycin or thapsigargin (Fig. Supplementary 1). This finding indicates that the lack of SELENON under hypoxic conditions induces a maladaptive ER stress response.

### 2.2 Both SELENON and ERO1 contribute to ER redox poise

ERO1 is a key enzyme in oxidative protein folding that also generates H_2_O_2_ [41], [46]. In order to compare the effect of induced ERO1 on ER redox poise in SELENON KD cells, a bicistronic plasmid expressing an ER-resident Flag-roGFP_iE, a modified green fluorescent protein with a metastable disulphide bond [22], and a hyperactive (it generates more H2O2) mutant of ERO1 * (C104A,C131A) was engineered and used to transfect WT and SELENON KD cells [14].

**Fig. 2:**
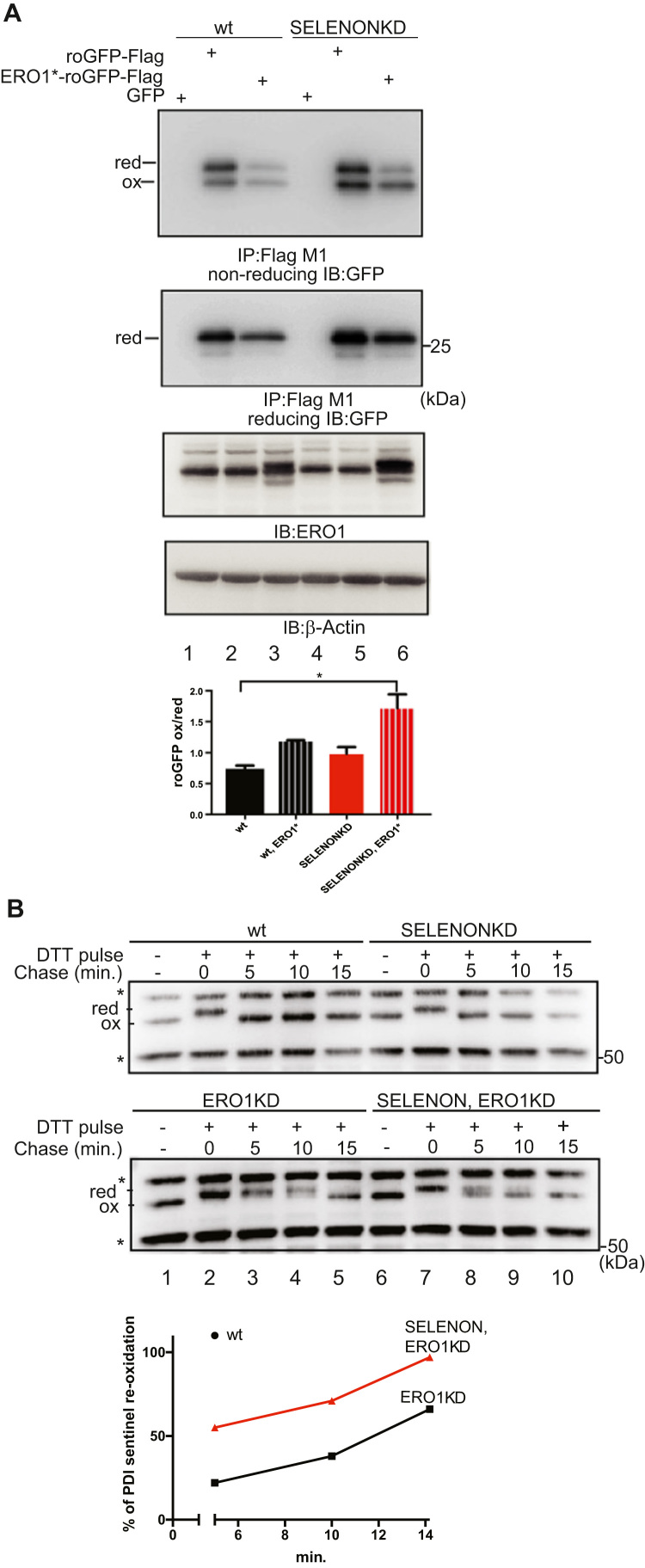
SELENON defends ER redox poise in presence of ERO1. A) Immunoblot of the ER-localised redox marker protein roGFP1_iE-Flag, FlagM1-immunoprecipitated from extracts of WT and SELENON KD cells, and resolved by means of non-reducing or reducing SDS-PAGE. The positions of the reduced (red) and oxidised (ox) forms of roGFP_iE are indicated in the non-reducing condition. Below, ERO1 immunoblot of the proteins from mock transfected, transfected with roGFP1_iE-Flag and with the bicistronic vector containing ERO1^*^ and roGFP1_iE-Flag. β-Actin was used as a loading control. Quantification (bottom) of the oxidised on reduced form of roGFP1_iE-Flag in non-reducing condition indicates the similar distribution of roGFP1_iE in the reduced and oxidised forms under basal conditions in both cell types, and the higher accumulation of the oxidised form in SELENON KD cells after ERO1* expression (n = 3). B) Immunoblot of endogenous PDI isoforms extracted from WT, SELENON KD, ERO1 KD and double SELENON/ERO1 KD cells after a reductive pulse with dithiothreitol, subsequent three washouts and resolved by means of non-reducing SDS-PAGE. The positions of the reduced and oxidised forms of the protein are indicated; the asterisk marks the position of redox-insensitive proteins that are insensitive to DTT and also react with this antibody (as already seen in [9]. Below, percentage of re-oxidation (with respect to the completely reduced DTT-treated PDI) during the three washouts of PDI. Note the faster re-oxidation of PDI in the double SELENON/ERO1 KD cells indicated by the quantification.

In SELENON KD cells, the lack of SELENON only slightly affected the steady-state distribution of oxidised to reduced roGFP (compare lane 2 with 5 of the first panel and the relative quantification of the oxidised to reduced roGFP in the bar graph of Fig. 2A), but ERO1* over-expression led to the redox marker becoming almost doubly oxidised, whereas the WT cells maintained the oxidised to reduced roGFP ratio better (compare lane 3 with 6 and the relative quantification of the oxidised to reduced roGFP in the bar graph of Fig. 2A), as shown by a non-reducing Flag Immunoblot suggesting that SELENON plays a role in defending against ERO1-induced hyperoxidation.

To investigate in further detail the link between SELENON and ER redox poise, we generated SELENON KD, ERO1 KD and double SELENON, ERO1 KD in HeLa cells (Fig. Supplementary 2) and followed the re-oxidation of a sentinel disulphide of a PDI family member after a pulse of the reducing agent dithiothreitol (DTT). No difference in the re-oxidation of the sentinel disulphide was detected between WT and SELENON KD, which is likely to be due to the fast kinetic of re-oxidation of this PDI member (compare WT vs SELENON KD in the upper panel of Fig. 2B). However, the lack of ERO1 substantially delayed the kinetic of re-oxidation, and therefore we could detect the faster re-oxidation of this PDI member in cells lacking both ERO1 and SELENON than in cells lacking ERO1 alone (compare ERO1 KD vs SELENON, ERO1 KD in the lower panel and the relative quantification of PDI re-oxidation of Fig. 2B). Taken together, these findings indicate an abnormally hyperoxidised ER redox state in cells both lacking SELENON and over-expressing ERO1, and suggest that SELENON may acts as a reductase.

### 2.3 Both SELENON and ERO1 affect SERCA-dependent Ca^2+^ entry into the ER

**Fig. 3:**
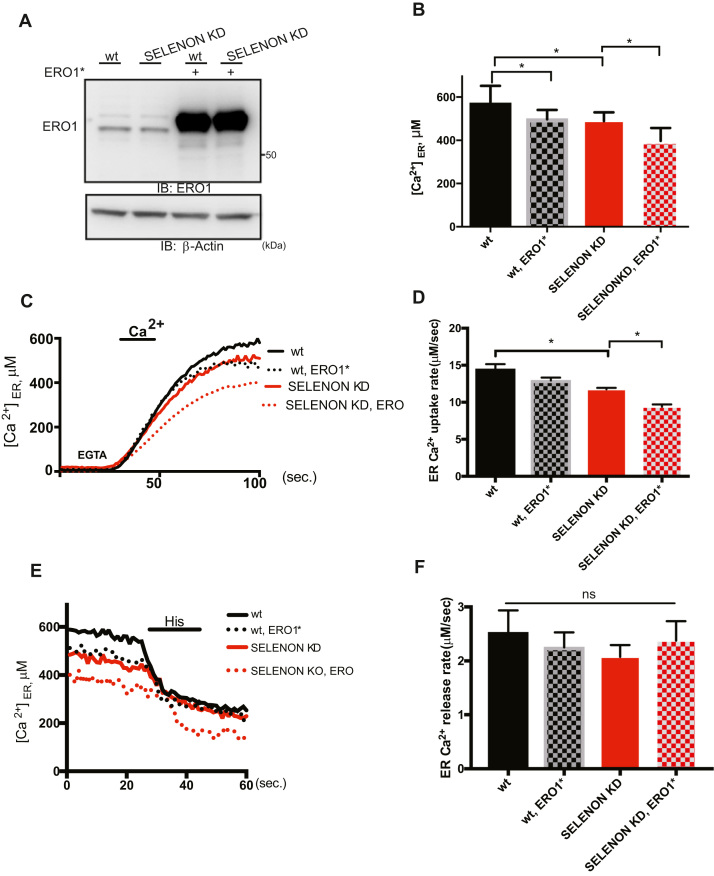
Lack of SELENON and high levels of ERO1 coordinatedly affect SERCA activity. A) ERO1 and β-Actin Immunoblots of WT and SELENON KD HeLa cells overexpressing ERO1*(C104A, C131A). B) WT and SELENON KD HeLa cells were co-transfected with ER aequorin and ERO1* and Ca^2+^ refilling of the ER was recorded. Bar graphs representing steady-state ER Ca^2+^ concentrations (n = 8). C) Measurements of ER [Ca^2+^] refilling. The traces are representative of eight independent experiments that gave similar results. D) Bar graphs representing the rate of Ca^2+^ uptake in the ER (n = 8). E) Measurements of ER [Ca^2+^] efflux after agonist stimulation (Histamin 100 mM). The traces are representative of eight independent experiments that gave similar results. F) Bar graphs representing measurements of ER [Ca^2+^] efflux after agonist stimulation (n = 8) (ns stands for not statistically significant).

As it has been previously shown that SELENON regulates SERCA-dependent Ca^2+^ entry [27], we investigated whether over-expressed ERO1* in WT and SELENON KD cells acts on the same pathway (Fig. 3 A). [Ca^2+^] was measured selectively in the ER of cells with a recombinant aequorin probe. The re-addition of Ca^2+^ to the medium rapidly increased [Ca^2+^] levels within the ER ([Ca^2+^]_ER_) up to a steady-state level of about 600 μM in WT cells, and 550 μM in SELENON KD cells. A significant difference was evident in [Ca^2+^]_ER_ of the cells over-expressing ERO1* and SELENON KD, that was significantly lower compared with SELENON KD (400 μM). A difference in intra-luminal [Ca^2+^] levels between the ER of the cells expressing ERO1* was not only observed at steady-state (Fig. 3 B), but also at the maximal rate of Ca^2+^ accumulation in the ER calculated at the beginning of ER Ca^2+^ refilling (i.e. when the rate mainly depends on SERCA activity) (Fig. 3C and D), thus suggesting a further decrease in SERCA activity when cells lack SELENON and over-express ERO1*.

Ca^2+^ efflux from the ER was also compared after the agonist stimulation (histamine) of IP3R1 but, as there was no between-group difference (Fig. 3E and F), the activity of the main ER Ca^2+^ release channel IP3R1 was unaffected by the lack of SELENON and the over-expression of ERO1*.

**Fig. 4:**
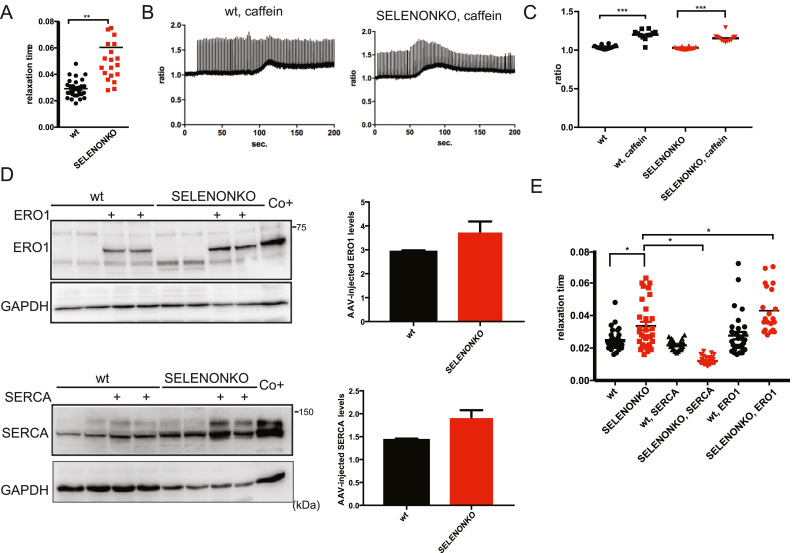
Lack of SELENON increases half-relaxation time by affecting SERCA activity. A) Half-relaxation time (milliseconds) in WT and SELENON KO muscle fibres from FDB muscle upon electrical stimulation with single twitches. B) Example of traces of caffeine-induced Ca^2+^ release in wildtype and SELENON KO fibres during electrical stimulation with single twitches. C) Maximal caffeine-induced Ca^2+^ release in WT and SELENON KO muscle fibres from FDB muscle in electrically stimulated fibres. D) Immunoblot for ERO1 and SERCA2 in WT and SELENON KO fibres mock-transduced or transduced with AAV-SERCA2A and AAV-ERO1. On the right, ERO1 and SERCA2 protein quantification after AAV infection. E) Half relaxation time in WT and SELENON KO fibres mock-transduced or transduced with AAV-SERCA2A and AAV-ERO1 upon electrical stimulation.

In order to investigate whether similar mechanisms are also active in skeletal muscle, we examined how the lack of SELENON affected isolated flexor digitorum brevis (FDB) fibres using the cytoplasmic Ca^2+^ sensor FURA-2. The half-relaxation time of the Ca^2+^ transients of electrically stimulated fibres, which indicates a decreased Ca^2+^ entry in the sarcoplasmic reticulum (SR) and therefore inversely correlates with SERCA activity, was higher in the SELENON KO mice than in their WT littermates (Fig. 4A), suggesting reduced SERCA activity in SELENON KO fibres.

Electrical stimulation in the presence of caffeine can be used to assess RYR-mediated Ca^2+^ release [2].

Therefore, to investigate whether SR Ca^2+^ release was also affected, we compared cytosolic calcium in FDB fibres from WT and SELENON KO mice after caffeine-induced stimulation (Fig. 4B). Interestingly, there was no difference in Ca^2+^ release between the WT and SELENON KO fibres, suggesting that the lack of SELENON does not affect RYR-mediated Ca^2+^ release (Fig. 4C).

In line with the results in cells, ERO1 over-expression in FDB muscle fibres obtained by injecting AAV2/1-ERO1 in FDB muscle (Fig. 4D, upper panel) further increased relaxation time in SELENON KO but was well tolerated in WT (Fig. 4E). In contrast, SERCA2A over-expression in FDB muscle fibres obtained by injecting AAV2/1-SERCA2A intramuscularly (Fig. 4D, lower panel) shortened relaxation time in SELENON KO FDB muscle fibres (Fig. 4E), indicating that the relaxation time nicely correlates with SERCA levels/activity.

These findings suggest that the lack of SELENON and the ERO1 over-expression in FDB muscle fibres selectively and cooperatively impair SERCA activity.

### 2.4 The lack of SELENON triggers diaphragm weakness associated with ER stress

Diaphragm dysfunction has recently been detected in pediatric patients with SELENON-related myopathies and occurs when the patients are still ambulant [6].

**Fig. 5:**
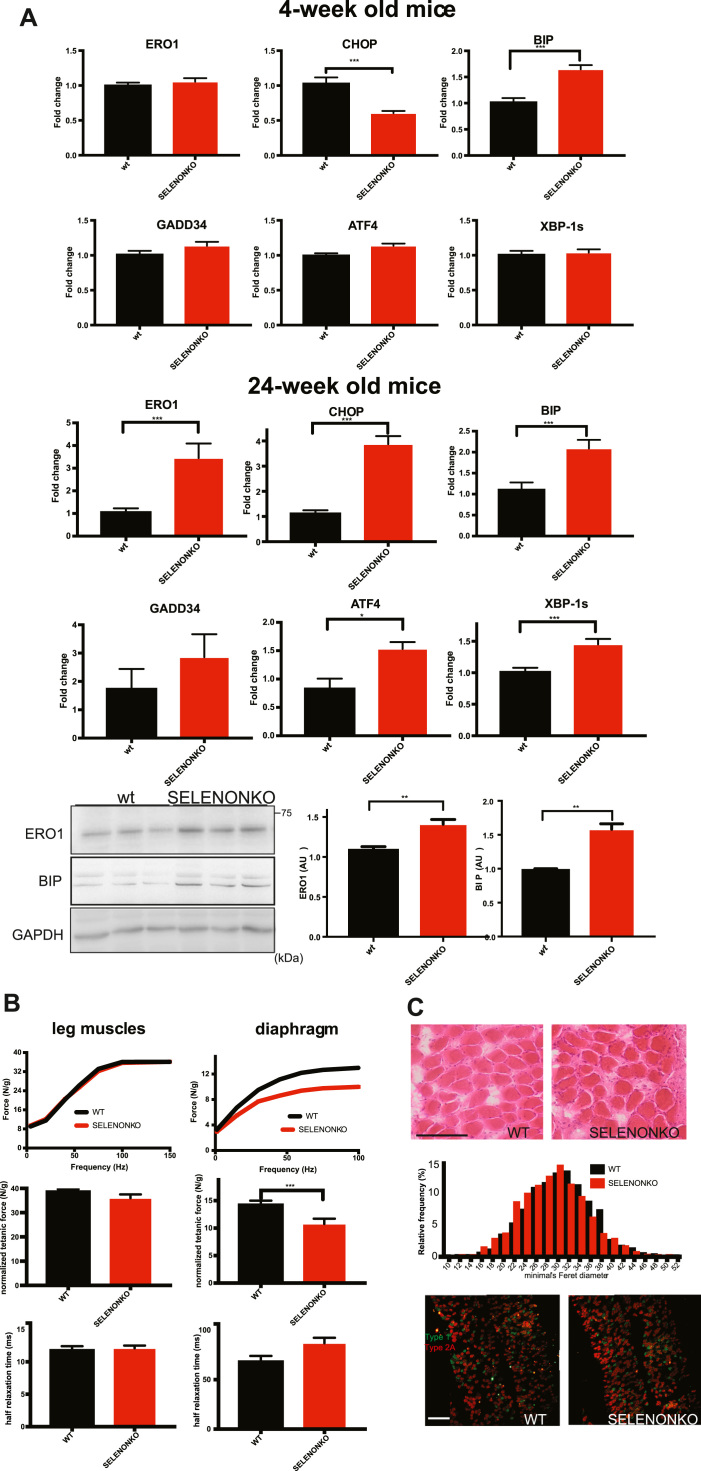
Diaphragm dysfunction in SELENON KO is associated with an exacerbated ER stress response. A) Semi-quantitative, real-time RT-PCR analysis of ER stress response markers in mRNA prepared from WT and SELENON KO diaphragms of 4- and 24-week-old mice (n = 12 for diaphragm of 4-week-old mice, n = 6 for diaphragms of 24-week-old). Bottom: ERO1 and BIP immunoblots and relative quantifications of the signals of proteins from 24-week-old mice, GAPDH was used as a loading control. B) Representative frequency curve, tetanic force and half relaxation time (stimulation frequency of 100 Hz) measured in vivo in the leg muscles (that mainly represents the force of the gastrocnemius muscle) (n = 12) and measured ex-vivo in strips of diaphragm (n = 20). C) Representative histology of H&E of diaphragms and minimal Feret's diameter (μm) of WT and SELENON KO diaphragms (n = 1200 fibres). Bottom: Representative fiber type immunostaining images in diaphragms using specific myosin heavy chain antibodies (Scale bars are 100 µm).

In order to understand whether this also occurs in the mouse model and is associated with an ER stress response we started by examining markers of the ER stress response in the SELENON KO diaphragm of four-week-old mice. There was a significant increase of the ER stress marker BIP in the diaphragm muscle of SELENON KO mice, suggesting an initial adaptive ER stress response. In contrast and as in cultured cells, there was a significant and marked increase of the maladaptive markers, CHOP and ERO1 in SELENON KO diaphragm muscle of twenty-four-week-old mice, suggesting a switch to a maladaptive ER stress response over time (Fig. 5A). Furthermore, *ex-vivo* force measurements on isolated strips of twenty-four-week-old old diaphragm muscle showed significant impairment in the normalised force of the SELENON KO diaphragm (not detected in leg muscles [35]) (Fig. 5B), which was accompanied by a trend towards longer relaxation time and without major morphological defects or fiber type switching (Fig. 5C). These findings suggest an overt maladaptive ER stress response in SELENON KO diaphragm muscle of twenty-four-week-old mice.

### 2.5 CHOP deletion rescues diaphragm dysfunction in SELENON KO mice

**Fig. 6:**
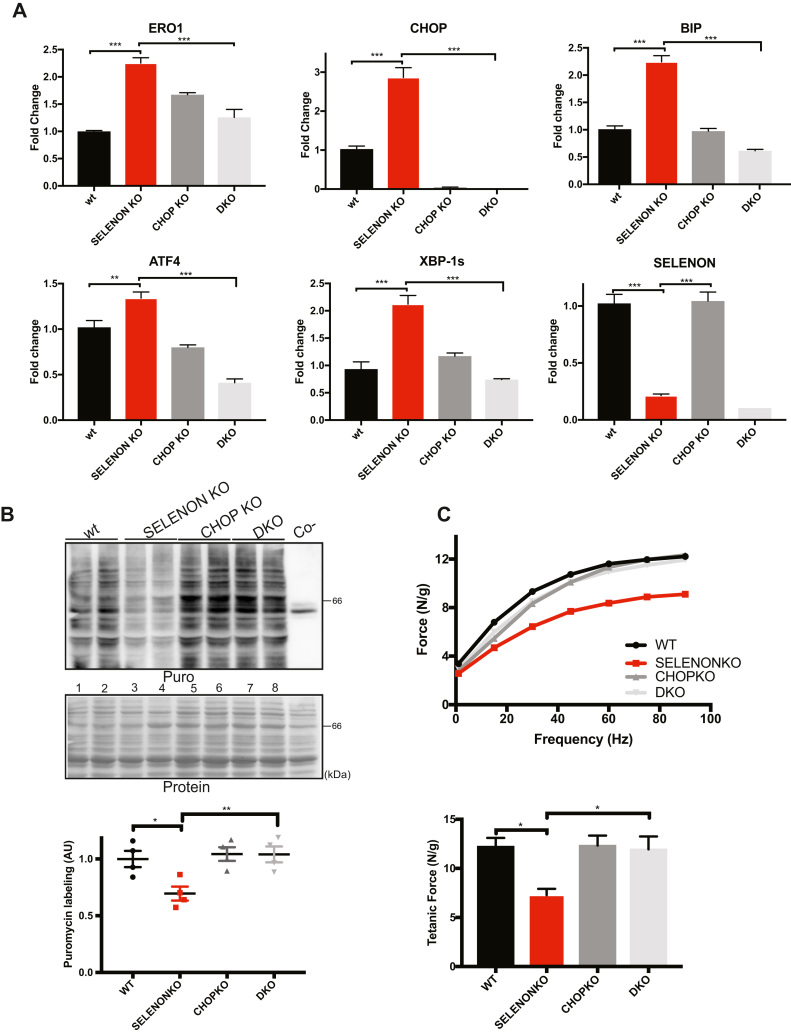
Deleting CHOP rescues diaphragm dysfunction in SELENON KO mice by reducing ERO1 levels. A) Semi-quantitative, real-time RT-PCR analysis of ER stress response markers of mRNA prepared from wild-type (WT) and SELENON KO, CHOP KO and double SELENON, CHOP KO (DKO) diaphragms (n = 8). B) Representative immunoblot of newly synthesised, puromycin-labelled proteins using an anti-puromycin antibody, and bar graphs of their signal in arbitrary units. C) Representative frequency curve and tetanic force measured ex-vivo on strips of diaphragm (n = 8).

Recent studies have shown that the genetic deletion of CHOP preserves tissue function after a pathological ER stress response [31], [39], [40]. Inspired by these studies and to understand whether the ablation of the excess of the CHOP-induced ERO1 during the ER stress response is beneficial to SELENON KO muscle we crossed CHOP KO mice with SELENON KO mice, and tested diaphragm function in WT, CHOP KO, SELENON KO, and double SELENON/CHOP KO (DKO) mice. The deletion of CHOP on a SELENON KO background lowered ERO1 levels and those of other ER stress response markers to those observed in WT mice (Fig. 6A).

As ER stress response promotes attenuation of protein translation, we examined if the rates of newly synthesised proteins were reduced in SELENON KO diaphragms. To do this we assessed the levels of protein translation in the diaphragms of WT, CHOP KO, SELENON KO and DKO mice using the SUNSET puromycin technique. In line with an attenuated ER stress response in DKO diaphragms, protein translation, which was decreased in SELENON KO diaphragms when compared to the WT (lanes 3 and 4 versus 1 and 2 and quantification of Fig. 6B), was completely restored in DKO diaphragms (lanes 3 and 4 versus 7 and 8 and quantification of Fig. 6B).

Importantly, the deletion of CHOP on a SELENON KO background completely recovered the reduced tension at all stimulation frequency and restored diaphragmatic tetanic force (Fig. 6C)

### 2.6 CHOP deletion rescues the prolonged relaxation time of SELENON KO limb muscle

**Fig. 7:**
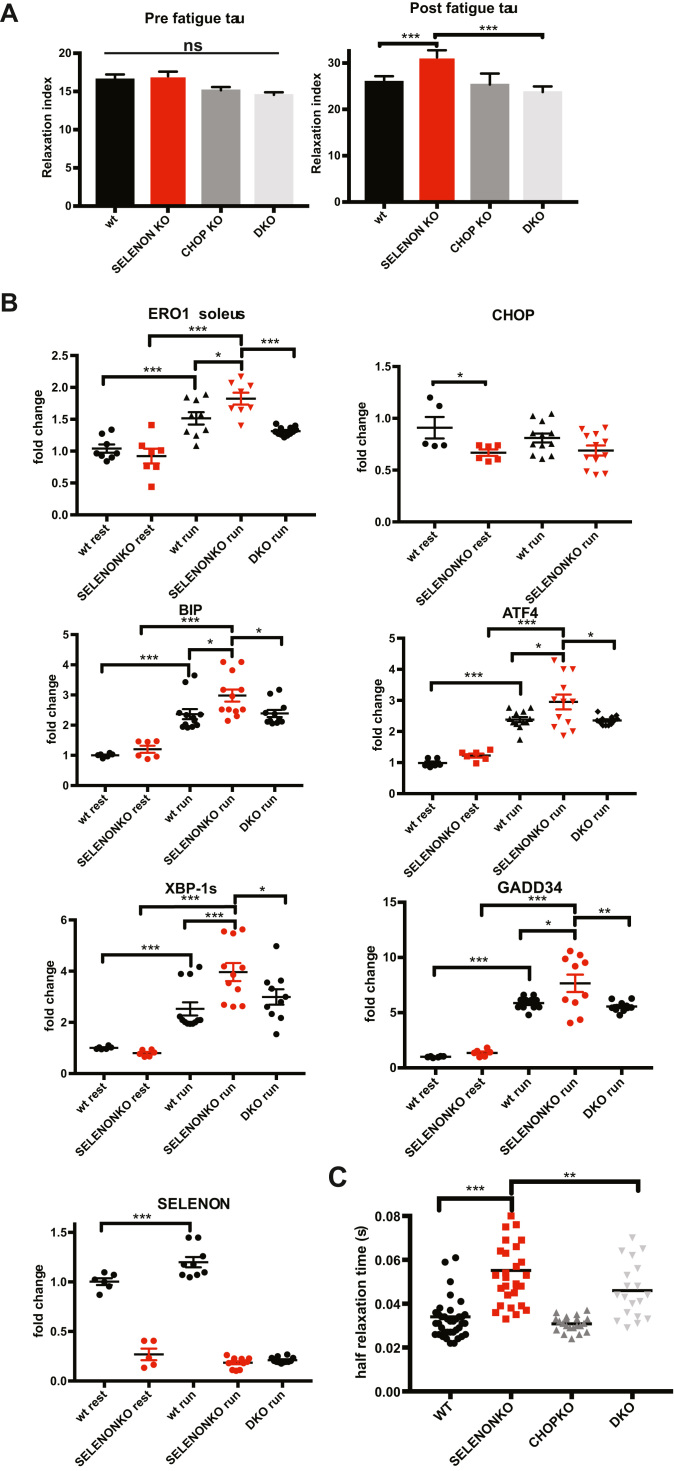
Deleting CHOP rescues defective limb muscle relaxation in SELENON KO mice by reducing ERO1 levels. A) Relative force reduction in limb muscles before and after 90 repeated maximal tetanic stimulations (n = 6). B) Semi-quantitative real-time RT-PCR analysis of ER stress response markers in mRNA prepared from WT, SELENON KO and DKO soleus of 24-week-old sedentary mice (rest) and mice after one bout of treadmill running (run). C) Half-relaxation time in isolated FDB fibres after electrical stimulation.

Although we and others have shown that the leg muscles of SELENON KO mice show no gross alterations in muscle histology, physiology or in the levels of the ER stress response markers [29], [36], [27], [35], we had detected a significant increase in the time constant of leg muscle relaxation after a series of tetanic stimuli, which represent an exercise mimetic that challenges SERCA activity [42] and requires an active SERCA [43]. Such a prolonged relaxation time was completely rescued in DKO mice, despite no changes in the resistance to fatigue (Fig. 7A).

To understand if SELENON function is important when activity levels rise during more physiological muscle activity, we investigated the expression of the ER stress response markers in soleus and gastrocnemius muscle isolated from mice after exhaustive running during which the muscle contractile activity significantly increases. Consistent with a maladaptive ER stress response that negatively influences SELENON KO muscle physiology, ERO1 and ER stress markers were more upregulated after running in SELENON KO soleus muscle than WT. Of note, ERO1 levels, and those of the other ER stress markers, were down-regulated in the DKO muscle after running (Fig. 7B and Fig. Supplementary 3) suggesting that in SELENON KO muscles and during muscle activity in a condition where SERCA activity is crucial, less ERO1 may be advantageous.

In accordance with a cause and effect correlation between a maladaptive ER stress response (i.e. that related to the CHOP-ERO1 branch) and a less active SERCA, the deletion of CHOP in a SELENON KO background shortened the relaxation time of FDB muscle fibres upon electrical stimulation, thus indicating a rescue of SERCA activity (Fig. 7C).

**Fig. 8:**
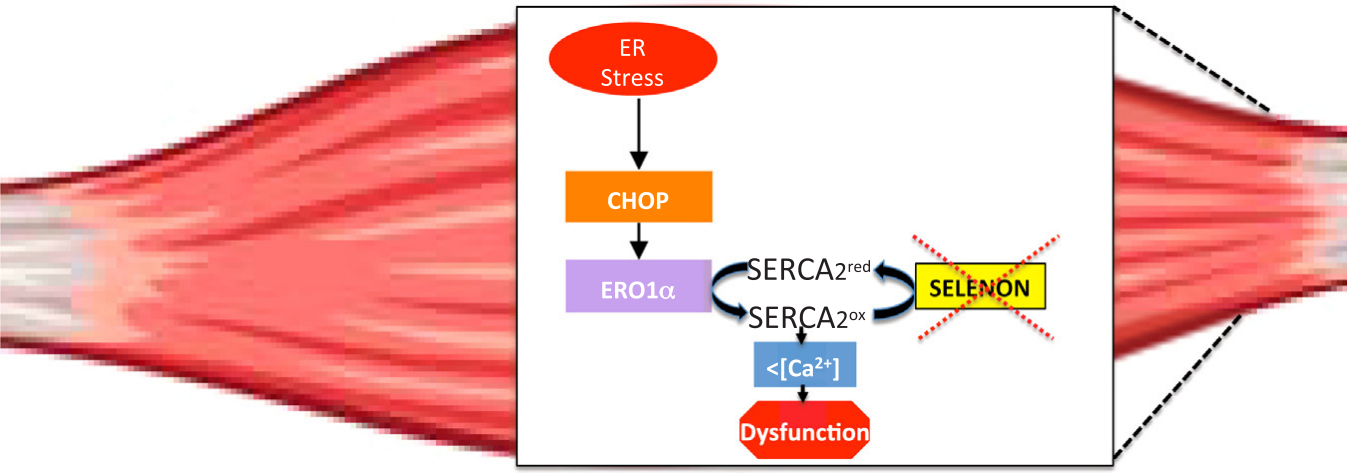
Maladaptive ER stress response in SELENON KO. The ER stress response is an ancient multi-dimensional signaling pathway initiated by ER stress and activated during muscle activity. Usually the ER stress response helps relieve cells from this stress so it serves as an important pro-survival pathway. However, high levels of ER stress persist if a “maladaptive ER stress response” fails to re-establish ER homeostasis and consequently cells are committed to dysfunction. Here we show that SELENON is part of an ER stress-dependent antioxidant response which, if it is missing, makes the CHOP/ERO1 branch of the ER stress response maladaptive quite likely by oxidizing and inhibiting SERCA2 in the highly active muscles.

Taken together, the deletion of CHOP from SELENON KO mice restored diaphragm weakness, the relaxation impairment of leg muscles after tetanic stimuli and SERCA-dependent Ca^2+^ re-uptake by attenuating ERO1 levels and those of the other ER stress response markers (Fig. 8), thus suggesting that the CHOP/ERO1 branch of the ER stress response is pathogenic in the active muscle of SELENON KO mice.

## 3 Discussion

SELENON-related myopathies are a group of congenital disorders arising from loss-of-function mutations in the SELENON gene that affect the muscle system, and lead to life-threatening respiratory malfunction, requiring assisted ventilation, with relative preservation of limb muscles and ambulation [23], [7]. No FDA-approved drugs are currently available for SELENON-RMs, so clarifying their pathogenic mechanisms is an important step towards developing a pharmacological treatment.

The presence of “minicores” (areas of mitochondrial depletion) in muscle biopsies is a hallmark of patients with homozygous loss-of-function mutations in *SELENON* and heterozygous mutations in ryanodine receptor 1 (*RyR1)* [10]. This overlapping phenotypic sign of the pathogenic mutations in *SELENON* and *RyR1* have prompted studies to characterise the functional interaction between SELENON and RYR1 as part of the pathogenic mechanism of SELENON-RM [18]. However, patients with recessively inherited RyR1-related core myopathies, unlike those with SELENON-related myopathies, lack severe respiratory impairment, and the different timing of the expression of SELENON and RyR1 raised questions about the physiological significance of the interaction between these two proteins [3], [17].

We recently used an unbiased proteomic approach to show that redox-active SELENON interacts with the SERCA2 Ca^2+^ pump and, in line with SELENON-mediated SERCA regulation, SELENON KO cells showed impaired SERCA activity and consequently lower ER Ca^2+^. Furthermore, SELENON levels are responsive to ER stress and are co-regulated with those of a mediator of the ER stress response, the disulphide oxidase ERO1, whose higher activity has been associated with negative outcomes [27], [20], [26]. The injection of ERO1-AAV into the gastrocnemius of SELENON KO mice, which is spared from any sign of myopathy, elicits a myopathy with a reduction in normalised muscle force. These results suggest a functional link between SELENON and ERO1 by which SELENON counteracts ERO1 activity [27], [35].

Interestingly, SELENON KO cells showed significant up-regulation of CHOP and its target ERO1 under hypoxic conditions, but there was no difference in their levels between SELENON KO cells and their WT counterpart after treatment with the known ER stressors, tunicamycin and thapsigargin (Fig. 1 and Sup Fig. 1). This suggests that SELENON function may be important in selected conditions such as hypoxia, and its absence elicits a maladaptive ER stress response characterised by the prominent up-regulation of CHOP and ERO1.

Here, cell studies uncovered a role for ERO1 in promoting a more oxidizing environment in the ER of SELENON-devoid cells. Likely, SELENON acts as a reductase (Fig. 2B) and the ER stress-mediated ERO1 over-expression hyperoxidizes the ER lumen of SELENON-devoid cells. Indeed, the enhanced rate of disulphide bond formation catalyzed by both ERO1 and the lack of SELENON affects the partitioning of luminal roGFP between its oxidised and reduced state (Fig. 2A), suggesting that SELENON may have evolved to become part of an ER stress-dependent anti-oxidant response. It is conceivable that the simultaneous lack of SELENON and overexpression of ERO1 shifts the equilibrium between the reduced and oxidised state of the two cysteine residues in the L4 luminal domain of SERCA2A, which previous studies have shown to modulate the ER Ca^2+^ concentration [21]. In line with this, and unexpectedly in view of studies indicating that SELENON plays a role in regulating RYR–dependent calcium release [18], the same condition of ERO1 over-expression in SELENON-devoid cells and muscle fibres affects the ER Ca^2+^ re-uptake governed by SERCA activity, without any major effect on Ca^2+^ release (Fig. 3 and 4).

SELENON KO mice resemble the phenotype of patients with SELENON-RM, insofar as they do not show major myopathic signs at the level of the leg muscles under normal cage conditions. However, our findings indicate that the relaxation kinetics of the leg muscle after tetanic stimuli (Fig. 7) as well as the force of the diaphragm muscle are impaired (Fig. 5 and 6) and that this correlates with higher levels of the maladaptive ER stress response markers CHOP and its target ERO1. This suggests a scenario in which, under conditions that elicit ER stress as the muscle activity, the absence of SELENON makes the ER stress response maladaptive by inducing ERO1, inhibiting SERCA activity and thus by weakening the excitation/contraction coupling machinery of highly active muscles (Fig. 8).

The reason for a major effect of SELENON-RM on the diaphragm and the relative preservation of the leg muscle is still object of study, but likely it is related to the high oxidative metabolism of the diaphragm in addition to its increased activity. Mitochondrial Ca^2+^ is directly influenced by ER Ca^2+^ levels through the mitochondria-associated membranes (MAMs) [12], [13], [8], where SELENON is enriched (EZ, personal communication), so it can be hypothesised that low ER Ca^2+^ levels reflect lower levels of mitochondrial Ca^2+^, influencing ATP production, and thus have a negative effect on diaphragm function [34]. However, in this particular model of SELENON deficiency, reduced pulmonary elasticity has also been reported leading to respiratory insufficiency [29]. In models of Chronic obstructive pulmonary disease (COPD) it is well established that long term exposure to hypoxic conditions leads to diaphragm dysfunction [19]. It is therefore tempting to speculate that a reduced pulmonary function in SELENON KO animals leads to hypoxia, which together to the muscle activity contributes to elicit a maladaptive ER stress response with a more prominent phenotype in the diaphragm.

Interesting recent studies have found that the genetic deletion of ERO1's transcription factor, CHOP, preserves tissue function during ER stress [31], [39], [40]. Therefore, we investigated whether CHOP and the CHOP-mediated induction of ERO1 during ER stress response are directly involved in SELENON-related myopathies by crossing SELENON KO with CHOP KO mice. The ablation of CHOP reduced ERO1 levels (Fig. 6 and 7), improved Ca^2+^ handling (Fig. 7), completely restored leg muscle relaxation after tetanic stimuli (Fig. 7), and diaphragm function (Fig. 6) in SELENON KO mice, indicating that the CHOP/ERO1 branch of the ER stress response is an important pathogenic cause of SELENON-RM.

This raises the intriguing possibility that chemical inhibitors of the ER stress response, when acting on the CHOP-ERO1 branch, might be used to treat SELENON-RM.

## 4 Experimental procedures

### 4.1 Animal experiments

All of the procedures involving animals and their care carried out at the Mario Negri Institute and the University of Padua were conducted as described by their institutional guidelines, which are in accordance with national (D.L. no. 116, G.U. suppl. 40, Feb. 18, 1992, No.8, G.U., 14 luglio 1994) and international laws and policies (EEC Council Directive 86/609, OJ L 358, 1 DEC.12,1987; NIH Guide for the Care and use of Laboratory Animals, U.S. National Research Council, 1996). The SELENON KO mice were purchased from the EMMA repository (SELENON<tm1.2Mred>/Orl), and the CHOP KO mice [45] (Stock No. 005530) from Jackson Laboratory.

### 4.2 SUNSET techniques

The rate of protein synthesis was analysed using the SUNSET method: the animals were starved for 30 min, injected with puromycin (0.040 μmol/g), and sacrificed 30 min after the injection.

### 4.3 FDB injection of AAV-ERO1 and AAV-SERCA2A

Six one-month-old WT and six one-month-old SELENONKO mice were injected with a total dose of 10^11^GC of ERO1-AAV2/1 or SERCA2a-AAV2.1 (Vector Biolabs) in the FDB muscle using a Hamilton syringe [27]. Equivalent doses of AAV2/1CMV-EGFP or equal volumes of PBS were injected into the contralateral muscle. The animals were sacrificed three weeks after the injection and the FDB fibres were cultured.

### 4.4 Muscle analysis

The muscles were mechanically disrupted using an Ultra Turrax homogeniser in RIPA buffer, for the SUNSET method a lysis buffer was used as in [38]. The insoluble material was isolated by means of clarification at maximum speed for 5 min, and the protein lysate was recovered and quantified using the BCA method.

The muscles were frozen in liquid nitrogen-cooled isopentane and fixed in formalin for morphological analysis, and cross-sections (8 µm) of isopentane-frozen muscle were stained with hematoxylin and eosin (H&E) for histological analysis.

### 4.5 Leg muscle force

Leg muscle force was measured as in [35]. Briefly, in vivo gastrocnemius muscle contractile performance was measured using a 305B muscle lever system (Aurora Scientific Inc.) in mice anesthetized with a mixture of Xylotine and Zoletil. Mice were placed on a thermostatically controlled table, the knee was kept stationary and the foot was fixed to a footplate, which was connected to the shaft of the motor. Contraction was elicited by electrical stimulation of the sciatic nerve. The torque developed during isometric contractions was measured at stepwise increasing stimulation frequency, with pauses of at least 30 s between stimuli. Force developed by plantar flexor muscles was calculated by dividing torque by the lever arm length (taken as 2.1 mm).

### 4.6 Diaphragm force

Diaphragm function was determined as described previously [4]. Briefly, force development measurements in the diaphragm were made by isolating small strips, mounting them between a force transducer and a micro manipulated shaft, and placing them in an oxygenated organ bath. Optimal length was determined through the length-tension curve at 100 Hz stimulation frequency. Tetanic force was determined after stimulation with trains of 500 ms once every two seconds at 100 Hz.

### 4.7 Fatigue protocol in vivo

Muscles were stimulated as described previously [24]. Briefly, two electrodes were placed on either side of the sciatic nerve and the common peroneal nerve was cut. The foot was fixed to a footplate and torque was measured using an Aurora Scientific 305 lever system. In order to induce muscle fatigue we applied 90 isometric tetanic contractions (100 Hz, 200 ms duration, once every second). We used a LabView analysis to determine the exponential decay of the force transient before and after the fatigue protocol.

### 4.8 Treadmill

For acute exercise studies we strictly followed the protocol in [16]. Briefly, mice were trained on an open treadmill (TSE-Systems) for two days. The third day mice started to run at a speed of 10 m/min for forty minutes, then the speed was increased of 1 m/min every 10 min for a total of 30 min and finally the speed was increased by 1 m/min every five minutes until the mice were exhausted. Exhaustion was defined as the point at which mice spent more than five seconds on the side of the lane. The muscles were isolated five hours after the treadmill running.

### 4.9 Real-time quantitative RT-PCR analysis

Total RNA was isolated from the cells and muscle tissue using the RNeasy Mini Kit (Qiagen) in accordance with the manufacturer's instructions. One microgram of total RNA was reverse-transcribed and analysed using the Applied Biosystems’ real-time PCR System and the ΔΔCt method. Relative gene expression in cells was normalised to beta-actin or cyclophilin, and relative gene expression in muscle was normalised to *GAPDH* mRNA levels.

The real-time primers were:

**Table:** 

mGADD34 F: GAGGGACGCCCACAACTTC R: TTACCAGAGACAGGGGTAGGT
mCHOP F: CCACCACACCTGAAAGCAGAA R: AGGTGAAAGGCAGGGACTCA
mBIP F: TCATCGGACGCACTTGGAA R: CAACCACCTTGAATGGCAAGA
mATF4 F: ATGGCCGGCTATGGATGAT R:CGAAGTCAAACTCTTTCAGATCCATT
mGAPDH F: CTGTGGTCATGAGCCCTTCC R: TGTTTGTGATGGGTGTGAACC
mERO1 F: CATACTCAGCATCGGGGGAC R: GAATGTGAGCAAGCTGAGCG
mSELENON F: GCTTTCCTGTAGAGATGATG R:GCCCCGCCGGAGTCCTTC
mbeta ACTIN F: CGCCTGAGGCTCTTTTCCAG R: TGCCACAGGATTCCATACCC
mXBP1–1 s F: GAGTCCGCAGCAGGTG R: GTGTCAGAGTCCATGGGA
hCyCLOPHILIN F: GACCCAACACAAATGGTTCC R:TTTCACTTTGCCAAACACCA
hERO1 F: GGCTTCTGGTCAAGGGAC R:TGCTTGCATGTAGGCCAGATA
hSELENON1 F: AGCTAACAGGGTCAACTCCC R:CGGCTGTCCAGTTTCGGAG

### 4.10 Vectors

The hyperactive hERO1A_C104A_C131A_pCDNA3_Sv40_roGFP1_iE plasmid encodes for SV40-driven SS_FLAG_roGFP1_iE (SS is an artificial signal sequence), and the CMV-driven hyperactive hERO1A_C104A_C131A.

The pCDNA3_Sv40_roGFP1_iE plasmid encodes for SV40-driven SS_FLAG_roGFP1_iE. The pCDNA3-hERO1A_C104A_C131A-Myc encodes for the human version of hyperactive ERO1 (C104A_C131A).

### 4.11 Antibodies

The following antibodies were used for Western blotting: monoclonal mouse anti-beta Actin from Santa Cruz, mouse anti-GAPDH from Sigma, mouse anti-puromycin from Merck Millipore, mouse anti-KDEL (to recognize BIP) from Enzo Life Sciences and polyclonal rabbit anti-ERO1α [47]. The following antibodies were used for Immunostaining: monoclonal BA-D5 (to stain type1 fiber) and SC71-S D5 (to stain type 2 A fiber) from Iowa Hybridoma bank.

### 4.12 CRISPR/CAS9 technology

SELENON KD, ERO1 KD and double SELENON, ERO1KD HeLa cells were generated with the CRISPR/CAS9 technology (OriGene), closely following the manufacturer's instructions.

Three different gRNAs were designed to target exon 2 of the human SELENON gene on chromosome 1. The gRNA that gave the best knock down was GAACTGGCGCTGAAGACCCT.

Three different gRNAs were designed to target exon 1 of the human ERO1 gene (*Homo sapiens* endoplasmic reticulum oxidoreductase 1 alpha). The gRNA that gave the best knock down was CCCCGGAGACAGCGGCACAG

Subconfluent HeLa cells were transfected in 6 cm Petri dishes with 5 μg of pCas-Guide DNA using OptiMEM medium (Gibco) and FuGENE HD transfection reagent (Promega) at a 1:3 DNA/reagent ratio as instructed in the manufacturer's manual. The transfection medium was replaced by DMEM/10% FBS 16 h after transfection, and 72 h after transfection the cells were collected, lysed and analysed for Cas9 expression by means of Western blotting. Semi-quantitative real-time PCR was used to determine the levels of SELENON and ERO1. A second round of transfection of ERO1 KD cells with pCas-Guide DNA to abolish SELENON levels was used to obtain double SELENON/ERO1 KD HeLa cells. The analyses were done on the cell pools because we could not obtain any viable single clone for the double SELENON/ERO1KD.

### 4.13 C2C12, SELENON knock-down, and lentiviral transduction

C2C12 cells were cultured in DMEM supplemented with 25 mM glucose and 10% fetal calf serum (FCS). SELENON was knocked down using Mission™ shRNA-encoding lentiviruses against mouse *SELENON* mRNA (SHCLND-NM_029100.2, Sigma) following the manufacturer's instructions [27], [35].

### 4.14 Ca^2+^ measurements

HeLa cells were grown in Dulbecco's modified Eagle's medium (DMEM) supplemented with 10% FCS in 75-cm^2^ Falcon flasks, seeded onto 13-mm glass coverslips, and allowed to grow to 50% confluence. At this stage, they were then transfected with endoplasmic reticulum (er) targeted aequorin (Aeq) chimeras together with various constructs, using the Ca^2+^ phosphate technique.

In order to ensure efficient reconstitution of the aequorin needed to produce the functional Ca^2+^-sensitive luminescent protein, the [Ca^2+^] in the lumen was reduced 36 h after transfection by incubating the cells for one hour at 4 °C in Krebs-Ringer buffer (135 mM NaCl, 5 mM KCl, 1 mM MgSO4, 0.4 mM KH2PO4, 5.5 mM glucose, 20 mM HEPES, pH 7.4) containing 5 μM coelenterazine, the Ca^2+^ ionophore ionomycin, and 600 µM EGTA. After incubation, the cells were extensively washed with KRB supplemented with 2% bovine serum albumin (BSA) and then transferred to the perfusion chamber before measuring their luminescence. The experiments were terminated by lysing the cells with 100 μM digitonin in a hypotonic Ca^2+^-rich solution (10 mM CaCl2 in H2O), thus discharging the remaining aequorin pool. The light signal was collected and calibrated into [Ca^2+^] values by an algorithm based on the Ca^2+^ response curve of aequorin at physiological conditions of pH, [Mg^2+^], and ionic strength as previously described [33], [5]. ER kinetics were analysed as described [25].

Ca^2+^ measurements in isolated single muscle fibres from the Flexor Digitorum Brevis (FDB) were performed as described previously [30]. Briefly, fibres were loaded with Fura-2 dye, and the Ca^2+^ transient after electrical stimulation (100 1 Hz was determined by the 360/380 nm ratio of the excitation wavelengths. Ca^2+^ release was induced by adding 20 mMolar of caffeine to the medium, while stimulating fibres electrically at 1 Hz.

### 4.15 Statistics

All results were expressed as mean ± SEM and are analysed using Prism 6 software (Graphpad). N was indicated in the figure legend except for dot plots. An unpaired *t*-test was used for the two-group analyses (Figs. 4A, 5A and 5B), a one-way ANOVA multiple comparison test was used for the analysis of three or more groups (Figs. 1, 2A, 3B, 3D, 3F, 4C, 4D, 4E, 6A, 6B, 6C, 7A, 7B, 7C, Sup. Fig. 1, Sup. Fig. 2 and Sup. Fig. 3). One asterisk was used for p < 0.05, two for p < 0.01, three for p < 0.001, and four for p < 0.0001.
