# Supplementary figures and images for: A maladaptive ER stress response triggers dysfunction in highly active muscles of mice with SELENON loss
Source: Redox Biol. 2018 Oct 26;20:354–66. doi: 10.1016/j.redox.2018.10.017 (PMC6223234; doi:10.1016/j.redox.2018.10.017)

A

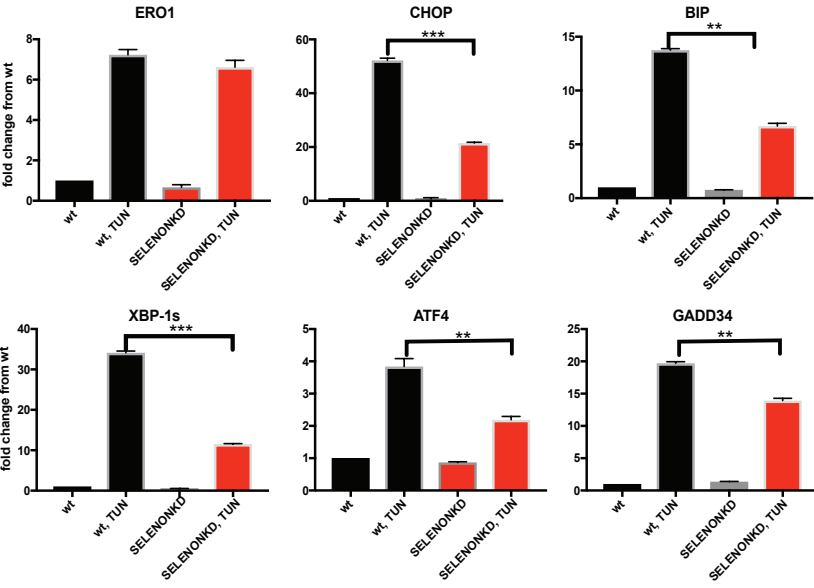

B

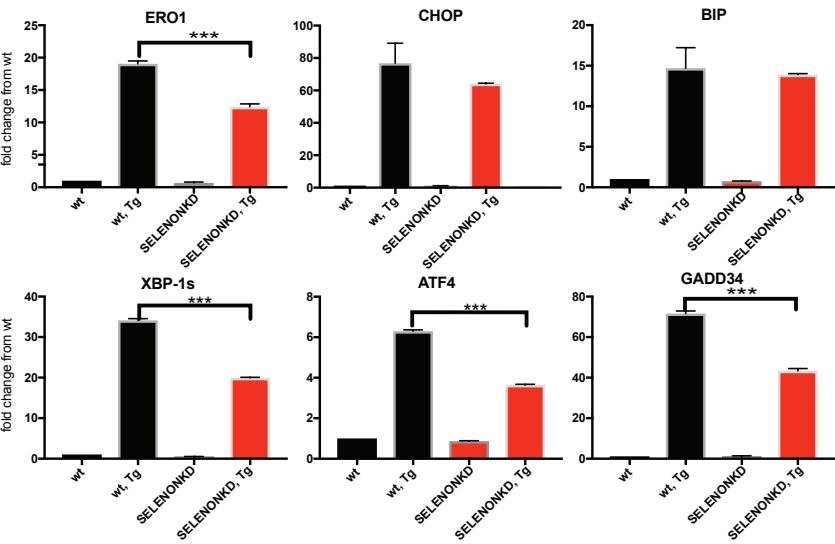

Supplement: Supplementary file 1 — Supplementary material [file mmc1.pdf]

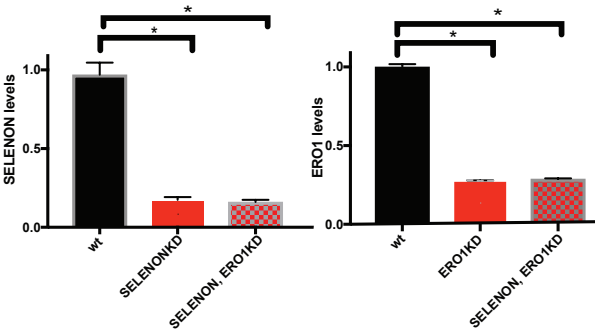

Supplement: Supplementary file 2 — Supplementary material [file mmc2.pdf]

ERO1 gastrocnemius

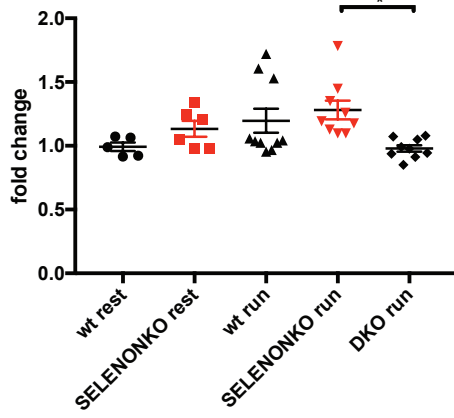

CHOP

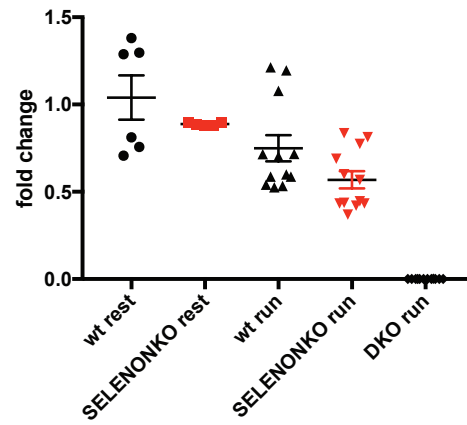

BIP

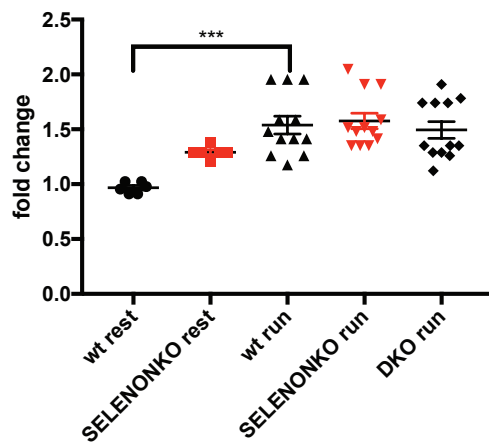

XBP-1s

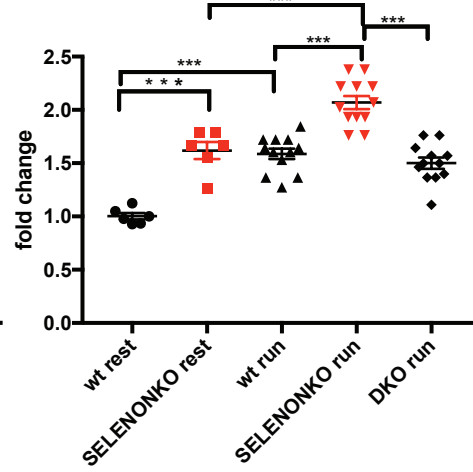

ATF4

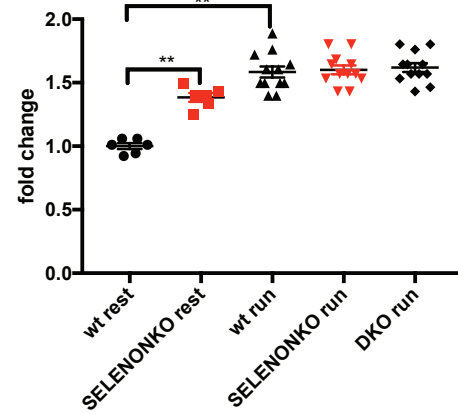

GADD34

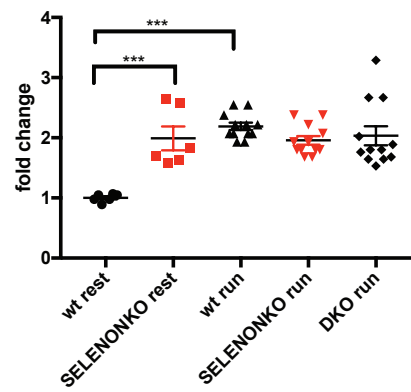

SELENON

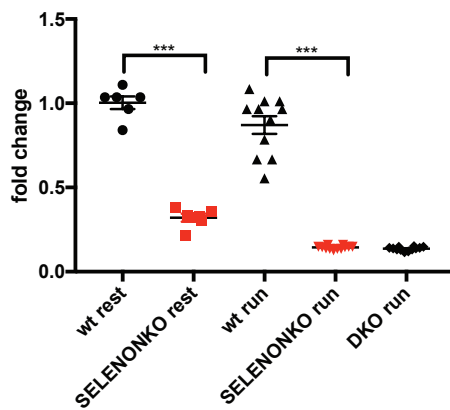

Supplement: Supplementary file 3 — Supplementary material [file mmc3.pdf]
