# Supplementary material for: A maladaptive ER stress response triggers dysfunction in highly active muscles of mice with SELENON loss
Source: Redox Biol. 2018 Oct 26;20:354–66. doi: 10.1016/j.redox.2018.10.017 (PMC6223234; doi:10.1016/j.redox.2018.10.017)
Supplement: Supplementary file 4 — Supplementary material [file mmc4.docx]

1. **Figure Supplementary 1**
2. A) Semi-quantitative, real-time RT-PCR analysis of ER stress response markers in mRNA prepared from WT and SELENON KD C2C12 after 12 hours exposure to tunicamycin (Tun, 5 μg/mL) (n=3).

B) Semi-quantitative, real-time RT-PCR analysis of ER stress response markers in mRNA prepared from wild-type (WT) and SELENON KD C2C12 cells after 10 hours exposure to thapsigargin (Tg, 0.5 μM) (n=3).

**Figure Supplementary 2**

SELENON KD, ERO1 KD and double SELENON/ERO1 KD HeLa cells were generated using the CRISPR/CAS9 knock out method, and the residual levels of SELENON and ERO1, due to the use of pools of cells, were tested by means of semi-quantitative, real-time PCR (n=3).

**Figure Supplementary 3**

Semi-quantitative, real-time RT-PCR analysis of ER stress response markers in mRNA prepared from WT, SELENON KO and DKO gastrocnemius of 24-week-old sedentary mice (rest) and mice after one bout of treadmill running (run).
